# Clostridiodes difficile Testing Protocol: An Effective Strategy to Decrease Over-Testing Patients for C. difficile Without Missing True Positive Infections

**DOI:** 10.7759/cureus.93174

**Published:** 2025-09-25

**Authors:** Sneha Kalluri, Daniel Cain, Abishek Volety, Ricardo Albarran-Anguiano, Franklin Obi, Alisha Jain, Shovendra Gautam

**Affiliations:** 1 Internal Medicine, Texas Health Resources Denton, Denton, USA; 2 Internal Medicine, Baylor Scott & White All Saints Medical Center, Fort Worth, USA; 3 Gastroenterology, HCA Medical City Fort Worth, Fort Worth, USA

**Keywords:** adult gastroenterology, clostridioides difficile infection, diarrheal illness, gastroenterology, infectious disease, quality improvement study, resource allocation, stool specimen

## Abstract

Objectives: Patients presenting with symptomatic diarrhea are frequently tested for *Clostridioides difficile* infection (CDI). However, increasing numbers of false positive results could lead to unnecessary testing and misuse of hospital resources. Additionally, physicians are often skeptical about the diagnostic stewardship protocols established by clinical institutions because of their uncertain negative predictive value and often err on the side of ordering the test despite the protocol (error of commission rather than error of omission). The objective of this study is to evaluate the implemented screening test protocol at Baylor Scott & White All Saints Medical Center in terms of its efficacy in mitigating the overuse of hospital testing resources while appropriately triaging true cases of CDI and the negative predictive value of the diagnostic stewardship protocol.

Methods: We performed a retrospective, observational study at Baylor Scott & White All Saints Medical Center, Fort Worth, between January 1, 2021, and January 1, 2022, where *C. difficile* testing was analyzed after implementation of our protocol.

Results: 443 patient encounters contained *C. difficile* stool testing orders. Of the 443 initial orders, 391 were confirmed to be canceled. Fifty-two orders were carried out, of which 7 were positive for *C. difficile* and 45 were negative. Of the 391 *C. difficile* tests canceled, 377 were due to the study protocol during the index encounter, and 14 were canceled for undocumented reasons. Of these 377 canceled orders, there were 12 false negatives due to a positive *C. difficile* result on the repeat test performed within 30 days of the index order canceled based on the protocol criteria, thus leaving 365 true negatives. The negative predictive value (NPV) of the protocol was 96.82% (365/377).

Conclusions: Our protocol proves effective in avoiding false positive hospital-related C. difficile infections, and at the same time, very effective in not missing out on the true positive *C. difficile* infections (excellent NPV). It is an important quality improvement initiative that can be implemented at any hospital.

## Introduction

*Clostridioides difficile* (*C. difficile*) is a bacterium that infects the gastrointestinal system, causing significant diarrheal illness with risk of progression to systemic *C. difficile* infection (CDI), sepsis, and bacteremia. It can be community-acquired (CA-CDI) or hospital-acquired (HA-CDI), and is typically associated with antibiotic use, gastric ulcer medications, and corticosteroids [1]. The annual disease burden of CDI in the United States in 2017 was 462,100 people, with an annual incidence of 143.6 cases per 100,000 people. Community-acquired CDIs account for 226,400 cases, with an incidence of 70.4 cases per 100,000 people. Hospital-acquired CDI accounted for 235,700 cases with an incidence of 73.3 cases per 100,000 people. The impact of this illness on the healthcare system has been estimated to be roughly $5 billion and results in over 15,000 deaths annually [2,3]. The impact of this infection has led to more stringent methods of testing and hygiene, but the indication for testing and appropriate allotment of resources is a balance that has not been standardized. The risk of false positive results and the subsequent cost of treatment in patients who have colonization is an ongoing concern that many institutions have sought to address [4].

The Infectious Disease Society of America (IDSA) and Society for Healthcare Epidemiology of America (SHEA) guidelines recommend that *C. difficile* infection should be considered in patients who are not taking laxatives and have three or more episodes of unexplained, unformed stools (consistency that scores a 6-7 on the Bristol Stool Scale) in 24 hours. Samples should only be collected if no other preexisting condition or medical history could account for the diarrheal illness. In children older than 12 months, testing is recommended only for those with prolonged diarrhea and risk factors. If these criteria are met and there is suspicion of CDI upon hospital presentation, then it is recommended that the patient receive multi-factorial testing unless the institution has another protocol in place [5]. The American College of Gastroenterology (ACG) also recommends multi-step testing algorithms, which include both a highly sensitive and a highly specific testing modality to help distinguish colonization from active infection (conditional recommendation, low quality of evidence) [6].

The purpose of this study is to evaluate the effectiveness of the Baylor Scott & White Health All Saints Medical Center CDI testing protocol and ensure it does not miss any CDIs due to the cancellation parameters of CDI testing outlined in the protocol.

## Materials and methods

The investigators used a retrospective, observational study at Baylor Scott & White All Saints Medical Center, Fort Worth, between January 1, 2021, and January 1, 2022, where CDI testing was analyzed after implementation of the protocol below. The investigators used the Epic EMR SlicerDicer software to identify all patient encounters with CDI testing orders. The patient charts evaluated were solely inpatient records during the previously stated time frame. Four hundred forty-three encounters with CDI orders were identified, and then investigators evaluated if infections were detected, if orders were canceled, the reason for cancellation, and the results of *C. difficile* assays done within 30 days of the original canceled order.

**Figure 1:**
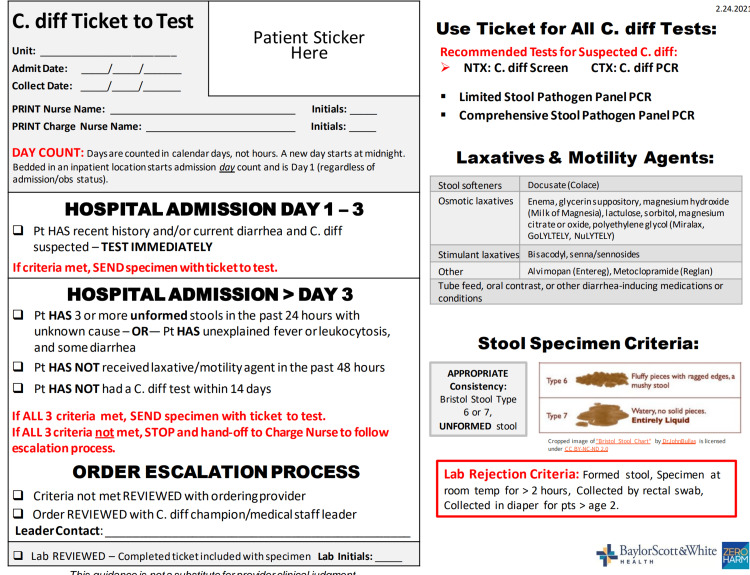
Example of the ticket to test to be completed for CDI testing

The criteria for HA-CDI were implemented after the 72-hour mark of a patient’s admission, with tests ordered prior to being within the margin for CA-CDI. The defined criteria to be tested included: 3 or more unformed (Bristol stool score 6-7) stools in the past 24 hours with an unknown cause or unexplained fever or leukocytosis in association with diarrhea, no laxative or motility agents within the last 48 hours, and no previous *C. difficile* testing within the last 14 days. If all three criteria were not met, *C. difficile* testing was canceled. Additionally, the lab checkpoint criteria for exclusion were defined as formed stool, specimen at room temperature for greater than 2 hours, rectal swab collection, or specimen collected from a diaper in patients older than 2 years of age. Specimen testing was assigned a testing ticket, as shown in the figure above (Figure 1), which provided concise, yet clearly defined protocol information for all healthcare staff involved. 

The CDI testing protocol at Baylor Scott & White All Saints Medical Center includes the provision that a stool sample for CDI evaluation can only be sent if the patient meets protocol criteria. If a provider ordered CDI testing in a patient who did not meet protocol criteria, these orders could be canceled after discussions between the primary provider or charge nurse and the ordering provider. In situations where there were disagreements, this would get escalated to the *C. difficile* physician champion, and a final decision regarding *C. difficile* testing would be made only after discussion between the primary provider and the physician champion. Additionally, the lab also verifies the appropriate stool consistency for CDI testing and could escalate the order for provider consideration of order cancellation if the stool consistency for testing criteria was not met. These orders can also be canceled by the primary team if symptoms resolve without any *C. difficile*-directed treatment or if another medical explanation for the diarrhea is found. The purpose of this study is to evaluate the negative predictive value (NPV) of the established protocol. In other words, the investigators want to evaluate if true *C. difficile* infections were missed due to testing being canceled based on the institution’s protocol.

Missed true infections were identified by using a positive *C. difficile* assay within 30 days of the original canceled order as the surrogate criteria for missed true clinical *C. difficile* infection during the index encounter. The NPV of the CDI protocol was then calculated for the evaluation of the protocol’s efficacy. Since there were no control arms or comparative groups used in this study, no p-value or additional statistical analysis was performed besides calculating the NPV.

## Results

**Figure 2:**
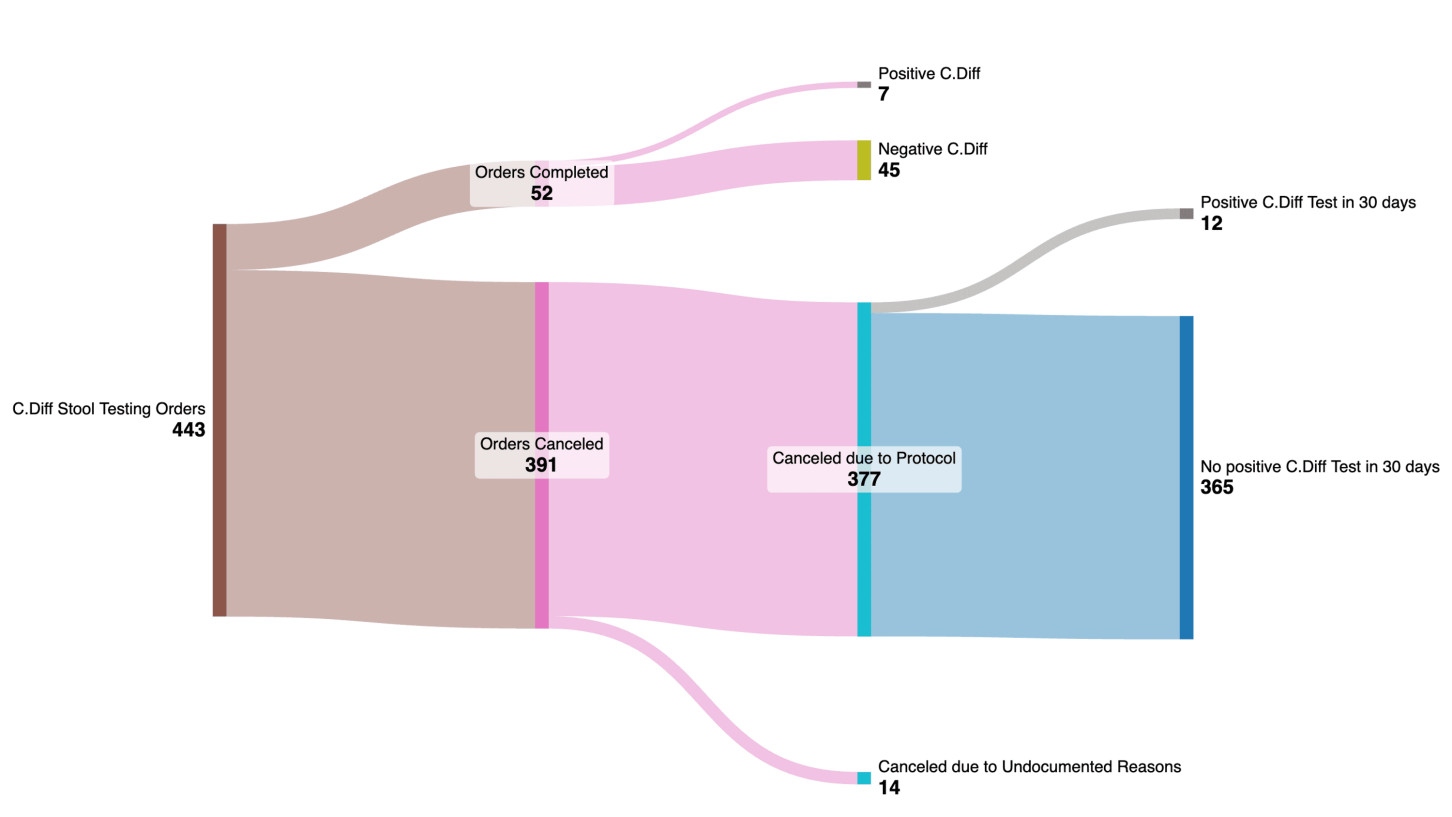
Sankey diagram demonstrating the Stool Testing orders and subsequent cancellations due to the implementation of the ticket-to-test protocol.

Four hundred forty-three patient encounters contained *C. difficile* stool testing orders. Of the 443 initial orders, 52 (52/443 (11.8%)) orders were completed of which 7 (7/52 (13.46%) of non-canceled orders, 7/443 (0.02%) of total orders) resulted as positive for CDI with 45 (45/52 (86.54%) of non-canceled orders, 45/443 (10.16%) of total orders) being negative for CDI. The remaining 391 orders (391/443 (88.2%)) were canceled. Three hundred seventy-seven of these canceled orders (377/391 (96.42%) of canceled orders, 377/443 (85.1%) of total orders) were due to the protocol parameters. Fourteen of the canceled tests (14/391 of (3.58%) of canceled orders, 14/443 (3.16%) of total orders) were due to undocumented reasons. Twelve patients whose index CDI test was canceled due to the protocol (12/377 (3.18%) of protocol-canceled orders, 12/443 (2.71%) of total orders) had a positive CDI result on the repeat test performed within 30 days of the canceled index order (Figure 2 and Table 1).

**Table 1:** CDI Order Data with Cancellation Sub-sets CDI: *Clostridiodes difficile* infection

Testing Subgroups	Number of Orders	Fraction (Percentage)
Total Clostridioides difficile Stool Testing Orders	433	433/433 (100%)
Orders completed	52	52/433 (12.01%)
Orders completed with a positive C. diff test	7	7/52 (13.46%)
Orders completed with a negative C. diff test	45	45/52 (86.53%)
Orders canceled	391	391/433 (90.30%)
Orders canceled due to undocumented reasons	14	14/391 (3.58%)
Orders canceled due to protocol	377	377/391 (96.42%)
Orders canceled due to protocol that did not meet lab testing criteria	116	116/377 (30.77%)
Orders canceled due to protocol with symptom resolution	162	162/377 (42.97%)
Orders canceled due to protocol with no stool sample collected	99	99/377 (26.26%)
Orders canceled due to protocol with positive C. diff test within 30 days	12	12/377 (3.18%)
Orders canceled due to protocol without positive C. diff test in 30 days	365	365/377 (96.82%) Data

Using these findings, the 377 canceled CDI orders due to the protocol and the 12 missed true positives found on subsequent 30-day testing were used to calculate the NPV of the CDI “Ticket to Test” protocol. This was done using 365 (377-12) true negatives (365/377 (96.82%) protocol canceled tests, 365/399 (91.48%) total canceled tests, and 365/443 (82.39%) of the total number of orders). The NPV of the protocol was 96.82%. The investigators concluded that with this result, the protocol demonstrates a high degree of accuracy and thus serves as an efficient diagnostic stewardship tool for prevention of unnecessary testing for *C. difficile* and concurrently helps minimize HA-CDI rates, an important safety metric for hospital systems nationwide.

## Discussion

The above results show that the protocol was efficient at determining which patients had a true infection with *C. difficile* and which patients did not. This decreased unnecessary testing and allowed many *C. difficile* orders to be canceled, saving hospital resources while preventing the over-testing of patients. The clearly defined criteria, as well as specimens sent for testing having an assigned testing ticket, as shown in the figure above (Methods, Figure 1), are what helped make this protocol a success.

**Figure 3:**
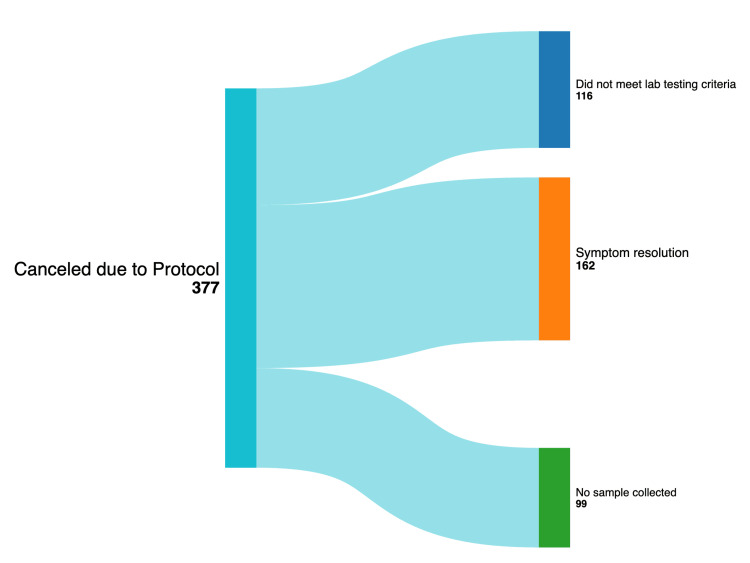
Sankey Diagram for Protocol Reasons for Canceled CDI Tests CDI: *Clostridiodes difficile* infection

In summary of the above results for orders canceled per the protocol, it showed that 85.1% (377/443) of *C. difficile* orders were canceled because of the implemented testing criteria. A further breakdown of which aspect of the cancellation protocol led to testing cancellations is as follows, with explanations for why each group was included in the protocol cancellation cohort. One hundred sixteen tests (116/377, 30.77%) were canceled due to testing criteria. 162 protocol canceled tests (162/377, 42.97%) were canceled due to the resolution of symptoms. This was considered an extension of the testing criteria, as it would mean there were fewer than three unformed stools in a 24-hour period. Ninety-nine tests (99/377, 26.26%) were canceled due to discharge prior to collection, or no sample was able to be collected (Figure 3). These cancellations were also considered to be extensions of the testing protocol, as it was once again an inability to collect stool and thus a decreased frequency of under three unformed stools within 24 hours.

To ensure that the exclusions were appropriate and not missing true cases of *C. difficile*, investigators reviewed the charts of the patients whose orders had been canceled. Based on the data above, the total negatives for the “Ticket to Test” protocol were 377 (i.e., the total number of indexed *C. difficile* orders canceled based on protocol criteria). Of these 377 canceled orders, there were 12 presumed false negatives (i.e., patients who had a positive *C. difficile* result on the repeat test performed within 30 days of the index order canceled based on the protocol criteria, and thus, there were 365 (377-12) true negatives). The 12 missed CDI infections were marked as “canceled due to protocol” in the respective patient charts, but the authors were unable to find data pertaining to which aspect of the CDI protocol the ordered test failed. This highlighted an area of improvement for the protocol documentation to ensure that those canceling the order document the specifics as to why, rather than a generalized reason. The NPV of the protocol was 96.82% (365/377). Per this metric, this protocol is effective in minimizing the overuse of testing while capturing true CDIs a majority of the time. Furthermore, the ticket to test is presented in a concise, easily available format to easily identify the parameters of testing by any provider involved in the patient’s care.

One limitation of this study is the possibility of missing information from the electronic health record system. For example, when chart-reviewing patients’ canceled orders, a positive *C. difficile* test within 30 days of initial discharge may not have been visible if the patient was later treated at a private or outlier facility. The second limitation of the study was the use of a positive *C. difficile* assay within 30 days of the original canceled order as the surrogate criterion for missed true clinical CDI during the index encounter. While this allowed for reasonable criteria to identify missed infection during the index encounter, it cannot be stated with absolute certainty that the CDI identified during the subsequent 30-day follow-up is rather a new infection and not a continuation of the hypothesized index infection. A third limitation of this study is that it limits the follow-up period for patients with canceled orders to 30 days. Risk of recurrent *C. difficile* infection can be increased for up to 8 weeks after initial infection, so some positive cases detected after the 30-day period may not have been included in the initial study [7,8]. Finally, the use of a control population prior to the protocol implementation would allow for evaluation of cost-benefit and comparison of CDI incidence pre- and post-protocol implementation. A future adjunct to this experiment may include further expansion of the record database along with an extended follow-up period.

## Conclusions

The findings of this study support the use of the above CDI testing protocol with an NPV of 96.82%. The minimal number of post-cancellation positives also supports the protocol's use. Further study with an additional 8-week re-test interval would be an additional step for further testing of the above protocol. A future prospective study with more robust cancellation documentation and additional centers would also be another way to further test the above protocol.
